# Supplementary material for: Assessing whether the best land is cultivated first: A quantile analysis
Source: PLoS One. 2020 Dec 10;15(12):e0242222. doi: 10.1371/journal.pone.0242222 (PMC7728207; doi:10.1371/journal.pone.0242222)
Supplement: S1 File — (DOCX) [file pone.0242222.s001.docx]

Assessing whether the best land is cultivated first: A quantile analysis

T. Brunelle^1^, D. Makowski^2,3^

^1^CIRAD, UMR CIRED, F-94736 Nogent-sur-Marne, France

^2^ Centre International de Recherche sur l’Environnement et le Développement, F-94736 Nogent-sur-Marne, France

^3^ UMR 211 INRAE AgroParisTech University Paris-Saclay 78850 Thiverval-Grignon France

Corresponding author

**Email:**  [thierry.brunelle@cirad.fr](mailto:thierry.brunelle@cirad.fr)

<https://orcid.org/0000-0001-5350-8332>

**Keywords**

Agricultural suitability; cropland; land rent theories; market accessibility.

**S1 Fig. Cropland distributions over quartiles of agricultural suitability in 15 world regions.**


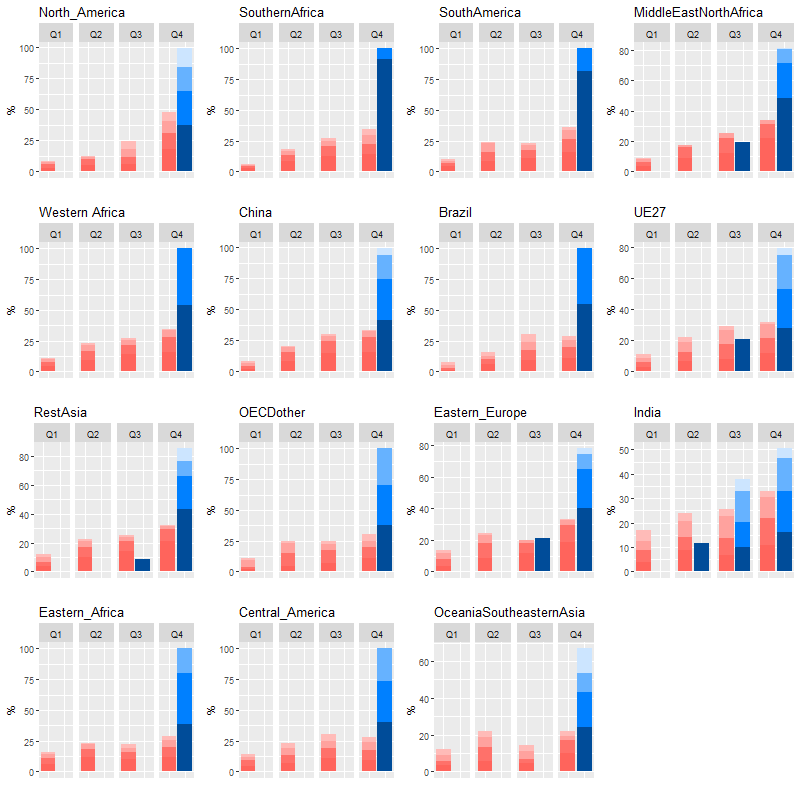


**S2 Fig. Cropland distributions over quartiles of market accessibility in 15 world regions.**

**
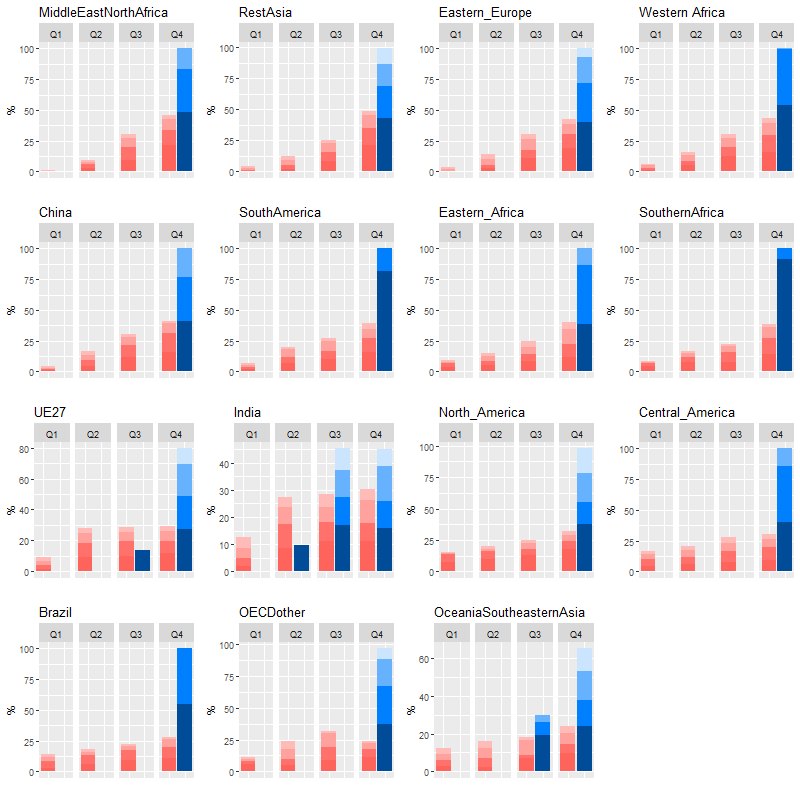
**

**S3 Fig. Crop fractions over quartiles of agricultural suitability in 15 world regions.**

**
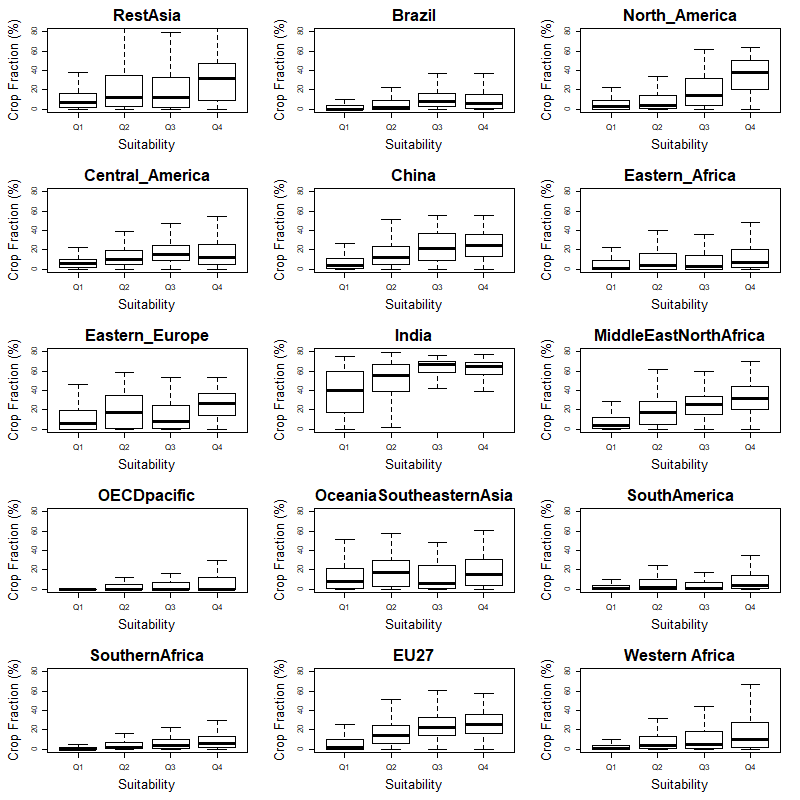
**

**S4 Fig. Crop fractions over quartiles of market accessibility in 15 world regions.**

**
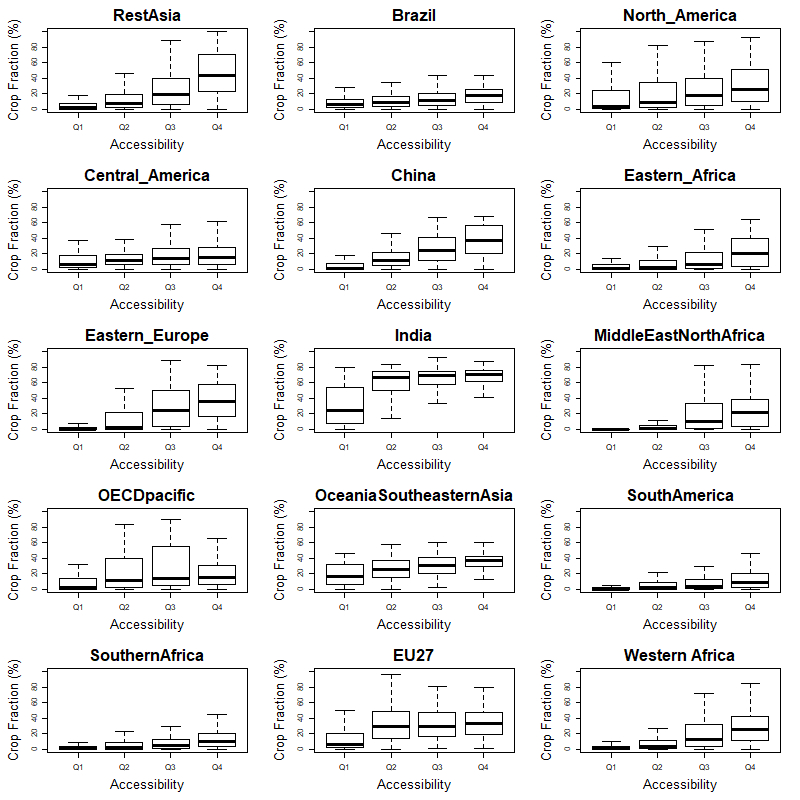
**

**S1 Table. Actual proportion of land area in quantiles of agricultural suitability.**

|  | Q1 | Q2 | Q3 | Q4 |
| --- | --- | --- | --- | --- |
| RestAsia | 23.5% | 23.9% | 27.0% | 25.6% |
| Brazil | 22.3% | 27.7% | 25.1% | 24.9% |
| North America | 23.0% | 25.8% | 24.3% | 27.0% |
| Central America | 24.5% | 24.1% | 25.8% | 25.6% |
| China | 24.7% | 25.8% | 24.6% | 24.9% |
| Eastern Africa | 24.9% | 23.5% | 26.0% | 25.6% |
| Eastern Europe | 24.8% | 24.1% | 25.8% | 25.3% |
| India | 22.6% | 25.5% | 22.3% | 29.5% |
| MiddleEast NorthAfrica | 26.0% | 24.3% | 24.7% | 25.0% |
| OCDEother | 24.4% | 23.9% | 23.2% | 28.5% |
| OceaniaSoutheasternAsia | 24.9% | 25.1% | 24.8% | 25.2% |
| South America | 23.6% | 24.8% | 26.9% | 24.6% |
| Southern Africa | 23.1% | 22.9% | 26.3% | 27.7% |
| UE27 | 24.1% | 25.6% | 24.8% | 25.5% |
| Western Africa | 24.5% | 24.1% | 25.5% | 25.9% |

**S2 Table. Actual proportion of land area in quantiles of market accessibility.**

|  | Q1 | Q2 | Q3 | Q4 |
| --- | --- | --- | --- | --- |
| RestAsia | 24.1% | 24.9% | 25.4% | 25.6% |
| Brazil | 25.7% | 25.3% | 24.5% | 24.4% |
| North America | 23.1% | 24.7% | 26.1% | 26.1% |
| Central America | 24.4% | 24.2% | 25.5% | 25.9% |
| China | 24.3% | 25.5% | 25.3% | 24.9% |
| Eastern Africa | 25.3% | 25.1% | 24.9% | 24.6% |
| Eastern Europe | 24.3% | 24.7% | 25.2% | 25.7% |
| India | 24.0% | 25.4% | 25.3% | 25.2% |
| MiddleEast NorthAfrica | 25.9% | 25.0% | 24.5% | 24.6% |
| OCDEother | 26.8% | 24.5% | 24.2% | 24.5% |
| OceaniaSoutheasternAsia | 25.4% | 25.3% | 23.7% | 25.6% |
| South America | 25.0% | 24.6% | 25.0% | 25.4% |
| Southern Africa | 25.5% | 25.0% | 24.9% | 24.6% |
| UE27 | 20.3% | 26.3% | 26.7% | 26.6% |
| Western Africa | 25.1% | 25.0% | 25.0% | 24.9% |

**S5 Fig. Suitability coefficients estimated by quantile regression for every 5^th^ percentile.**
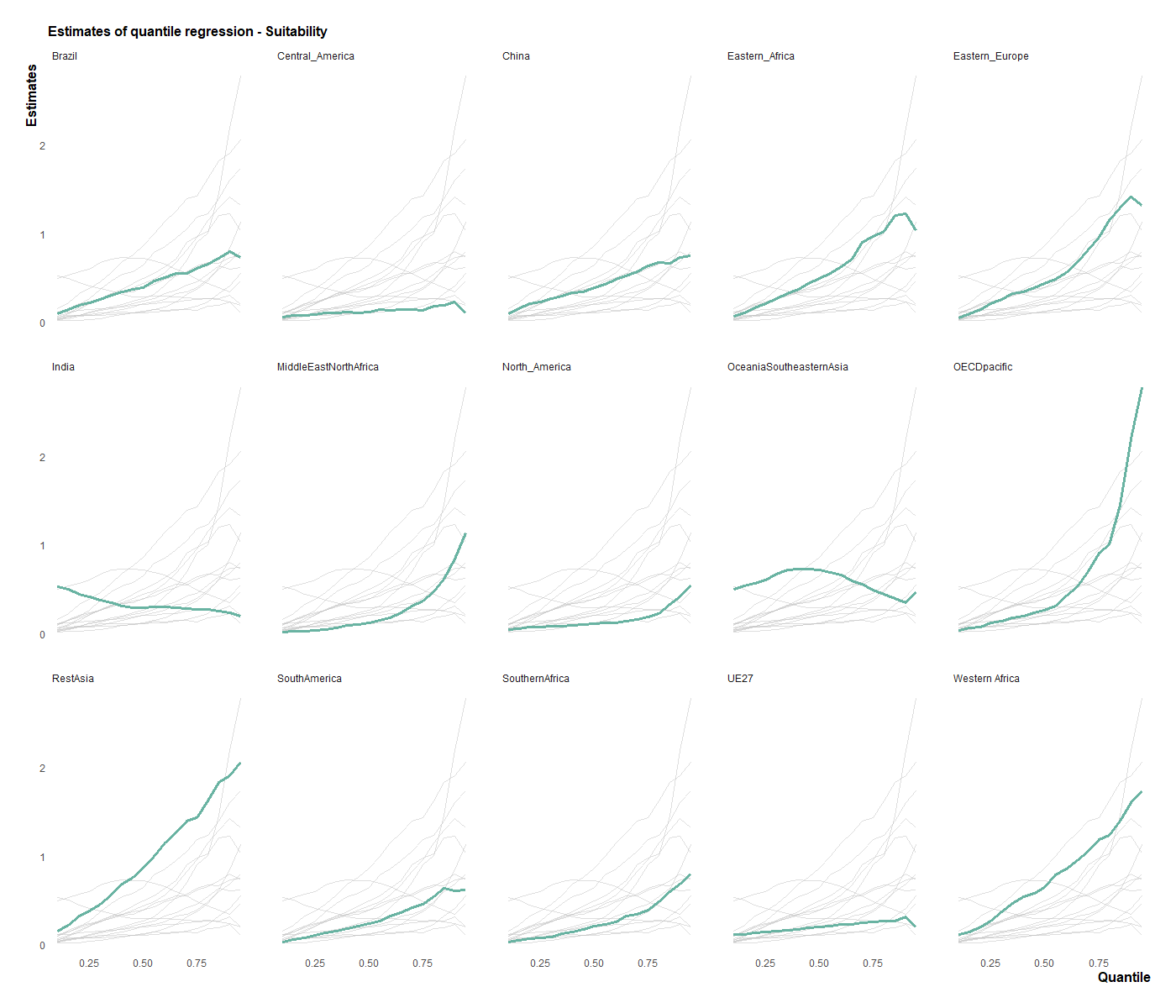


**S6 Fig. Accessibility coefficients estimated by quantile regression for every 5^th^ percentile.**
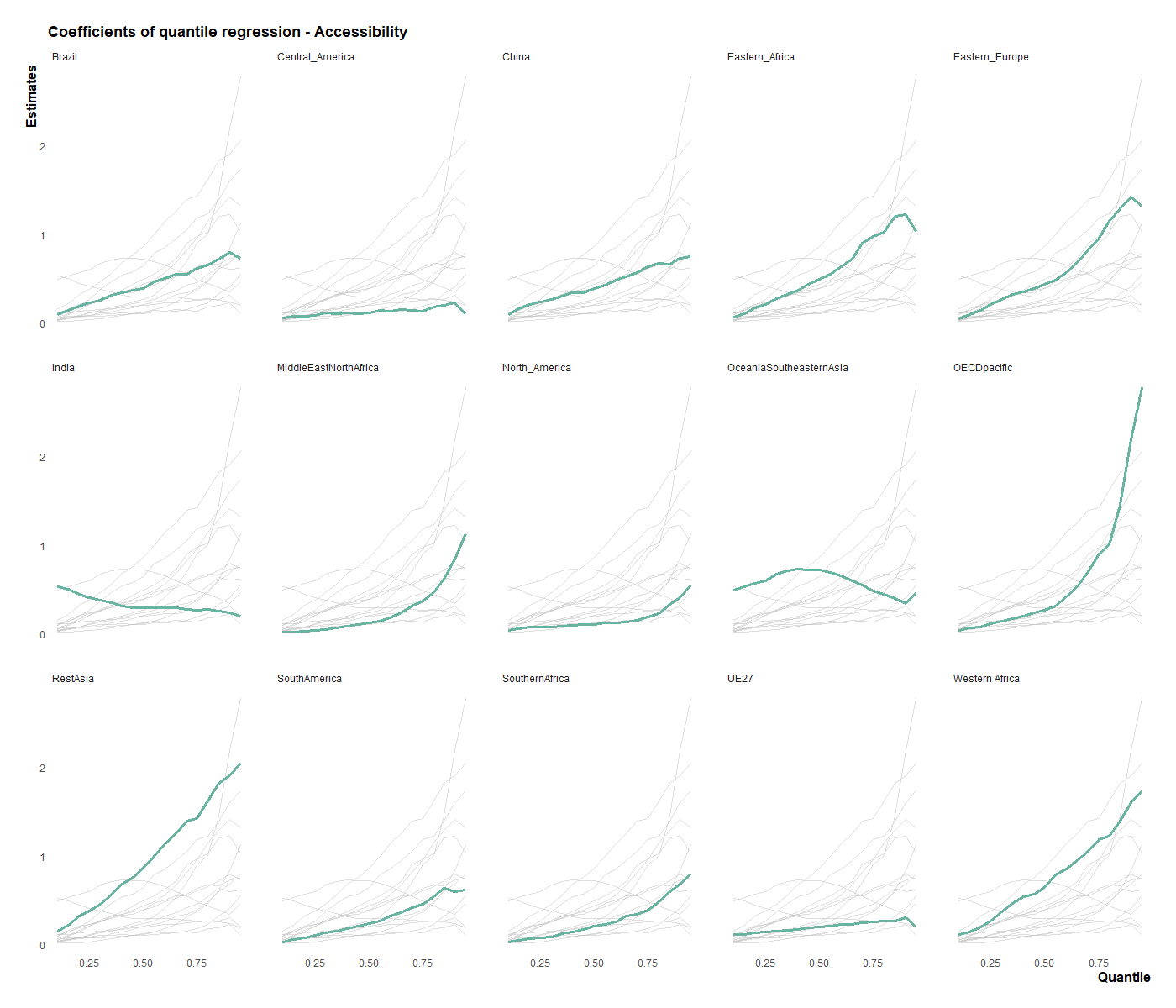


**S7 Fig. Interaction coefficients estimated by quantile regression for every 5^th^ percentile.**
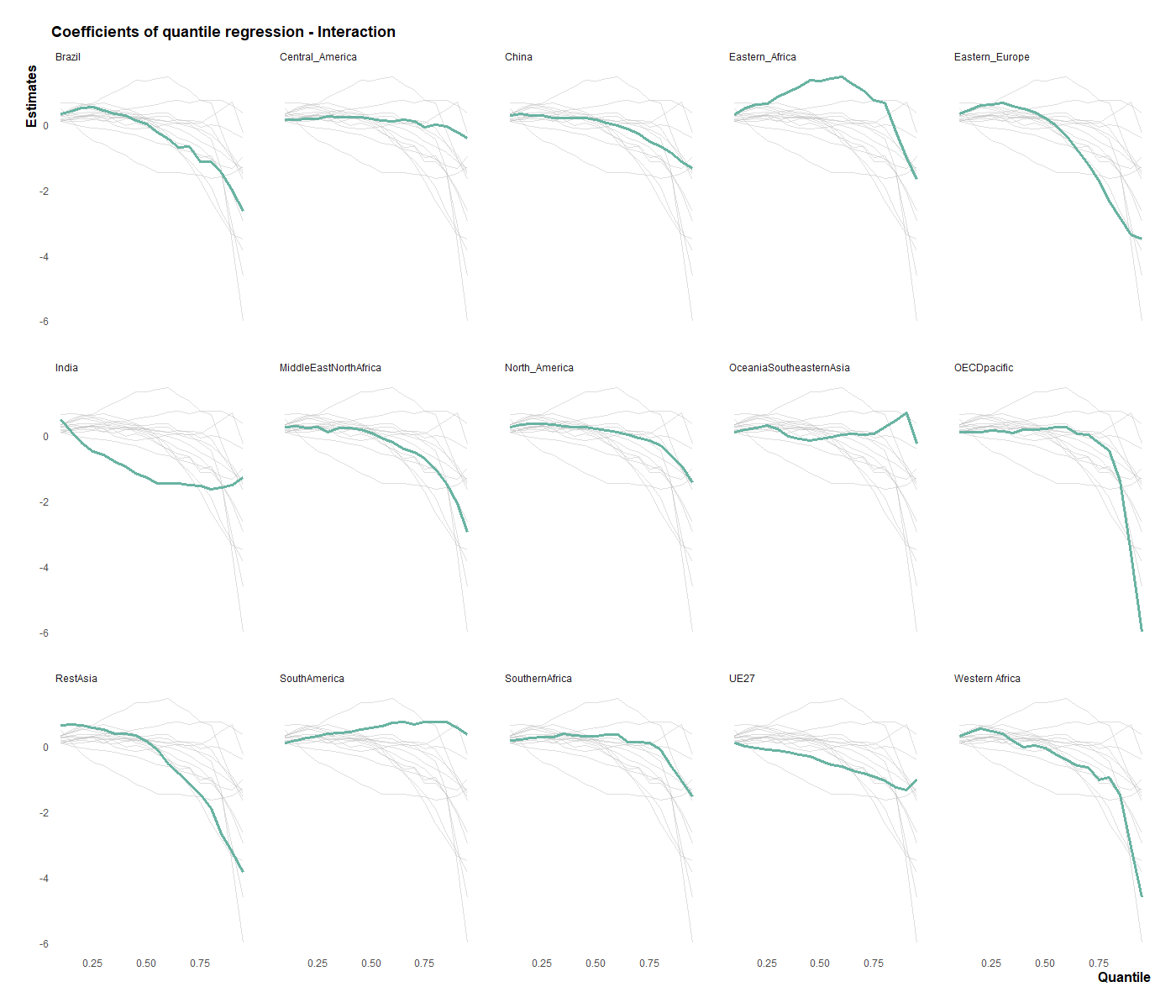


**S3 Table. Spearman coefficient correlation between suitability and accessibility (1^st^ column) and suitability and influence (2^nd^ column).**

|  | Suitability/Accessibility | Suitability/Influence |
| --- | --- | --- |
| RestAsia | 0.42 | 0.43 |
| Brazil | 0.18 | 0.24 |
| North America | 0.49 | 0.53 |
| Central America | 0.07 | 0.04 |
| China | 0.62 | 0.64 |
| Eastern Africa | 0.11 | 0.13 |
| Eastern Europe | 0.59 | 0.67 |
| India | 0.42 | 0.42 |
| MiddleEast NorthAfrica | 0.46 | 0.47 |
| OCDEother | 0.25 | 0.30 |
| OceaniaSoutheasternAsia | 0.13 | 0.16 |
| South America | 0.14 | 0.17 |
| Southern Africa | 0.16 | 0.05 |
| UE27 | 0.51 | 0.40 |
| Western Africa | 0.27 | 0.32 |

**S4 Table. Results of the ordinary least square regression of the ratio of quantile regression estimates suitability vs accessibility (denoted “RatioEstimates”) on the ratio of coefficients of variation suitability vs accessibility (denoted “RatioCV”). Estimates at the 25^th^ percentile are considered for India, and at the 75^th^ for the other regions.**

Call:

lm(formula = RatioEstimates ~ RatioCV)

Residuals:

| Min | 1Q | Median | 3Q | Max |
| --- | --- | --- | --- | --- |
| -0.91851 | -0.46956 | -0.2981 | 0.09435 | 2.77025 |

Coefficients:

|  | Estimate | Std. Error | t value | Pr(>\|t\|) |
| --- | --- | --- | --- | --- |
| (Intercept) | -0.3349 | 0.5219 | -0.642 | 0.53215 |
| RatioCV | 2.7555 | 0.8649 | 3.186 | 0.00716** |

Signif. codes: 0 ‘***’ 0.001 ‘**’ 0.01 ‘*’ 0.05 ‘.’ 0.1 ‘ ’ 1

Residual standard error: 0.9461 on 13 degrees of freedom

Multiple R-squared: 0.4384, Adjusted R-squared: 0.3952

F-statistic: 10.15 on 1 and 13 DF, p-value: 0.007159

The Moran test for spatial auto-correlation reveals that both our dependent and independent variables are spatially autocorrelated. Thus, the spatial autocorrelation of our response variable (crop fraction) is probably caused by our autocorrelated predictors (suitability and accessibility). In this case, it is not relevant to remove this effect from our predictors because our objective is precisely to estimate the effects of suitability and accessibility on the crop fraction. However, in order to avoid any unintended effect resulting from a residual autocorrelation, all p-values and confidence intervals of our estimates were computed using a non-parametric bootstrap procedure with 1,000 replications. The corresponding confidence intervals are shown in S8, S9 and S10 Figs of S1 File and do not indicate any spurious effect.

S8 Fig. Estimated effects of agricultural suitability for the third quartiles of cropland fraction in 15 regions. Estimates were produced by quantile regression. Confidence intervals (CI) are constructed using bootstraps method with 1000 replications.


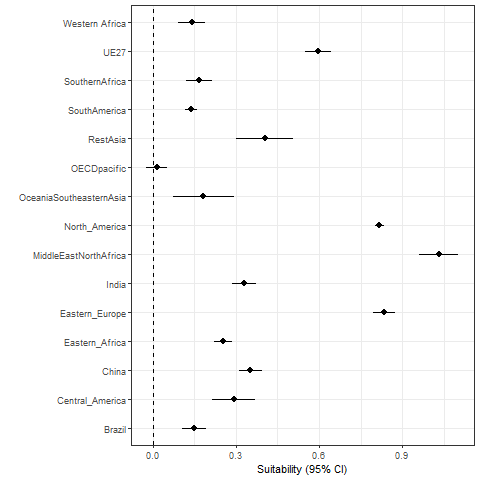


S9 Fig. Estimated effects of market accessibility for the third quartiles of cropland fraction in 15 regions. Estimates were produced by quantile regression. Confidence intervals (CI) are constructed using bootstraps method with 1000 replications.


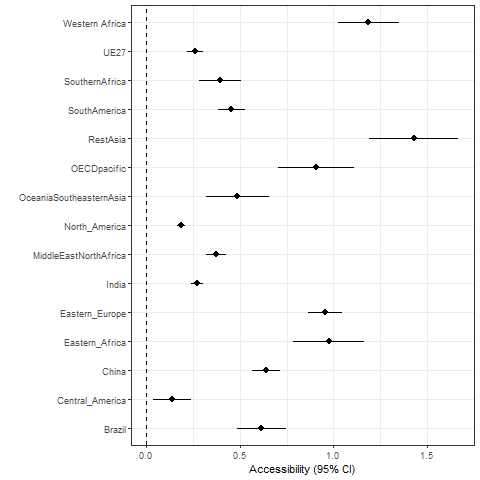


S10 Fig. Estimated effects of interaction between agricultural suitability and market accessibility for the third quartiles of cropland fraction in 15 regions. Estimates were produced by quantile regression. Confidence intervals (CI) are constructed using bootstraps method with 1000 replications.


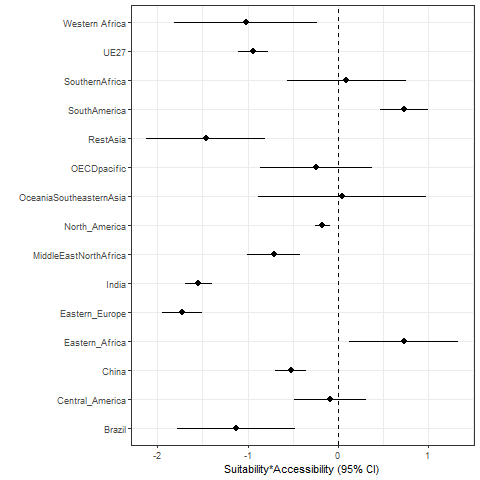


S11 Fig. Estimated effects of agricultural suitability (Panel A), market accessibility (Panel B), and interaction suitability/accessibility (Panel C) for the first and third quartiles of cropland land fractions in 15 regions. Estimates were produced by quantile regression using the Erb et al. land use dataset (Erb et al., 2007). The first and third quartile represent the 25% least and most cultivated land. Colors indicate the levels of statistical significance for each region.


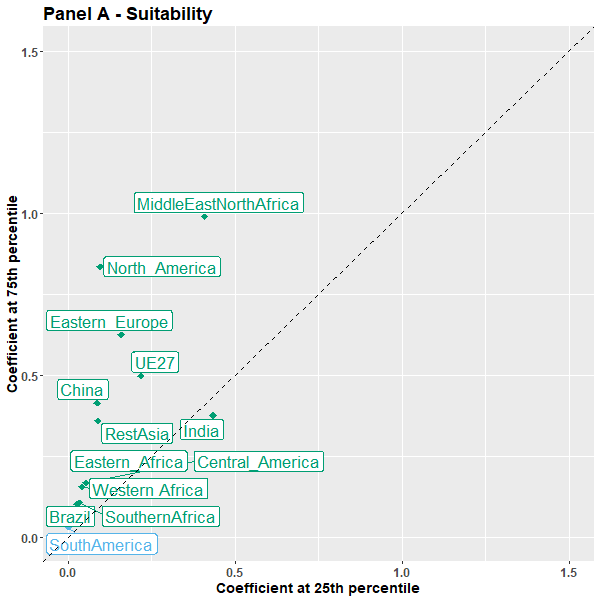

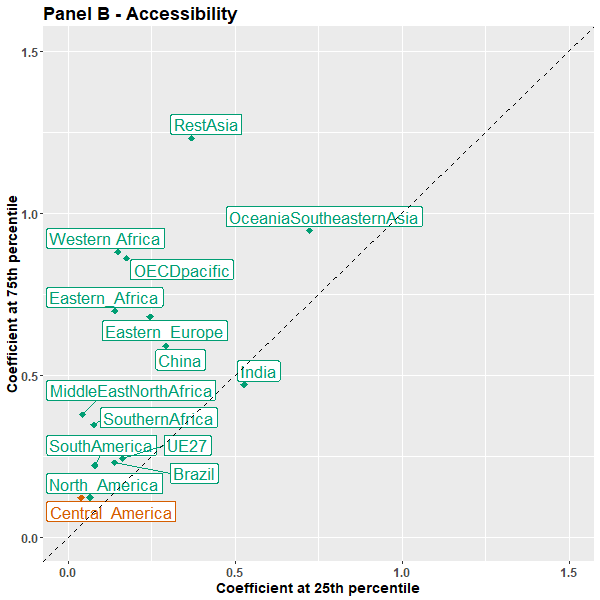


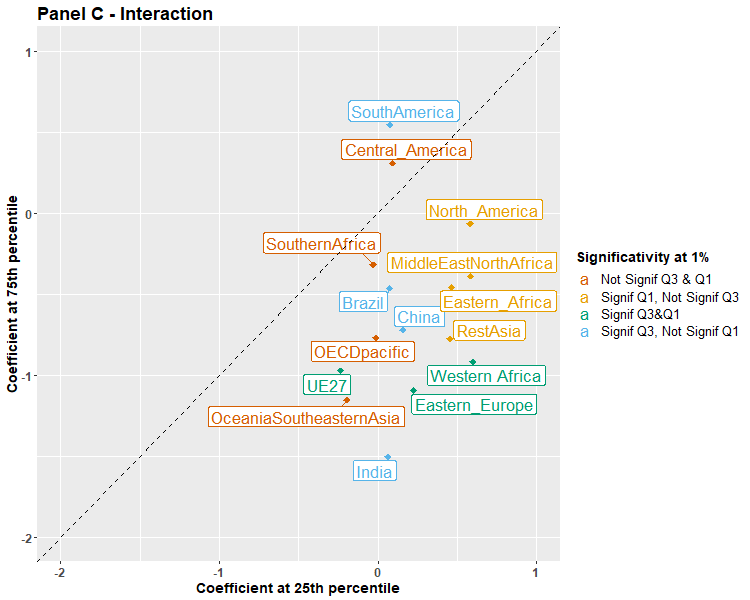


S12 Fig. R1 of quantile regression of two univariate models linking crop fraction to agricultural suitability (y-axis) and market accessibility (x-axis) using the Erb et al. land use dataset (Erb et al., 2007). The highest R1 between the first and third quartiles of the distribution of crop fraction (Q1 and Q3) is reported. Values are computed at the third quartile for the regions labelled in black, at the first quartile for the regions labelled in blue, and at the third quartile regarding suitability (suit.) and the first quartile regarding accessibility (access.) for the region labelled in red


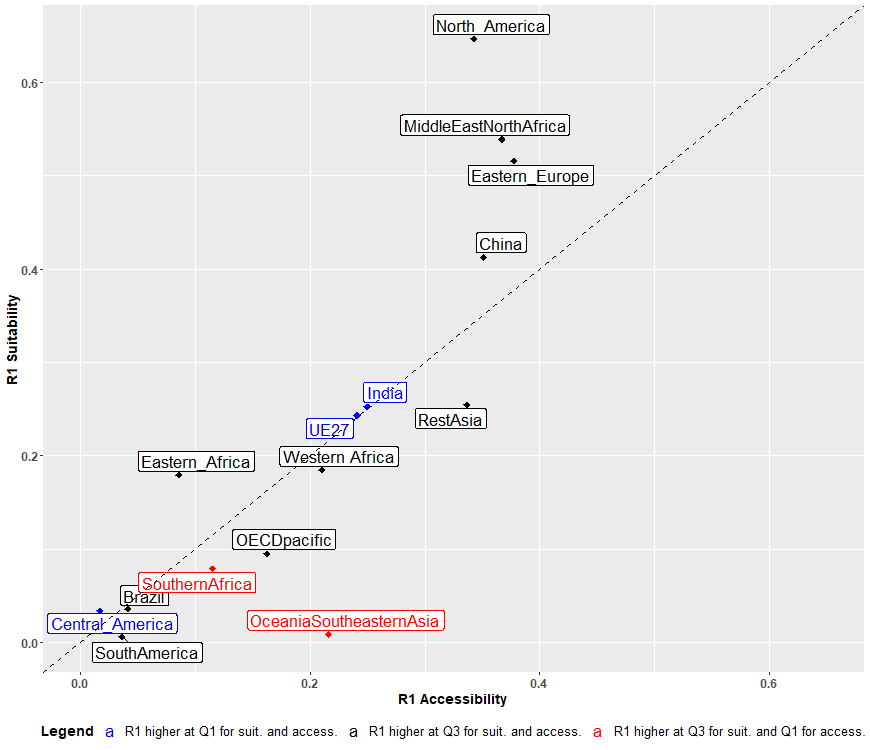


S13 Fig. Estimated effects of agricultural suitability (Panel A), market influence (Panel B), and interaction suitability/influence (Panel C) for the first and third quartiles of cropland land fractions in 15 regions. Estimates were produced by quantile regression using the Goldewijk et al. land use dataset (Klein Goldewijk et al., 2017). The first and third quartile represent the 25% least and most cultivated land. Colors indicate the levels of statistical significance for each region.


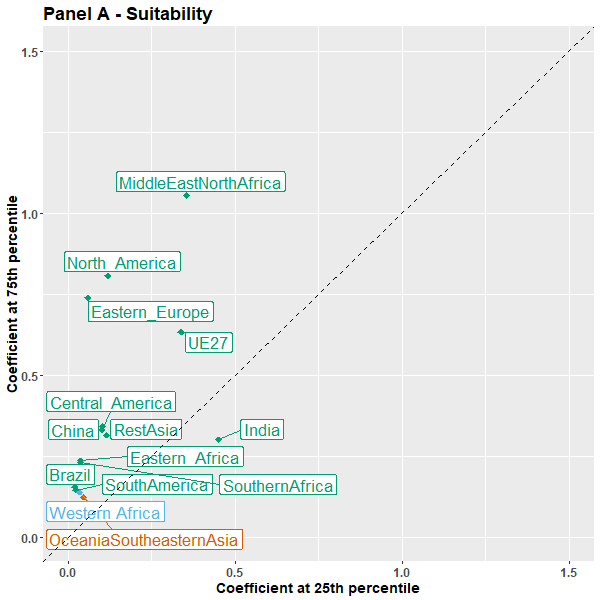

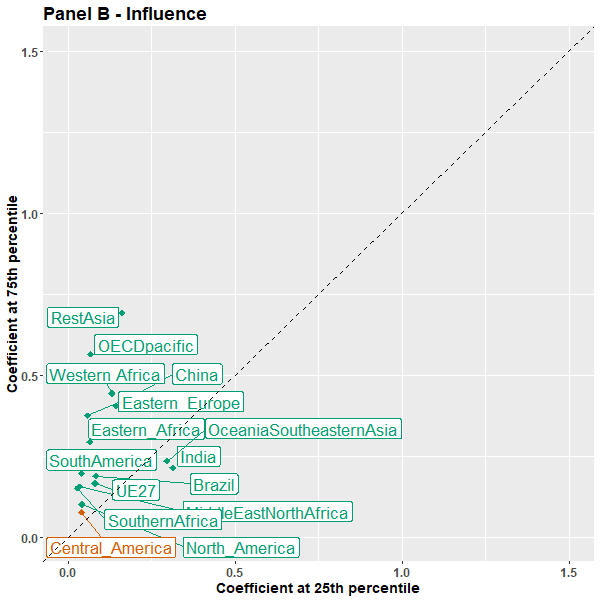


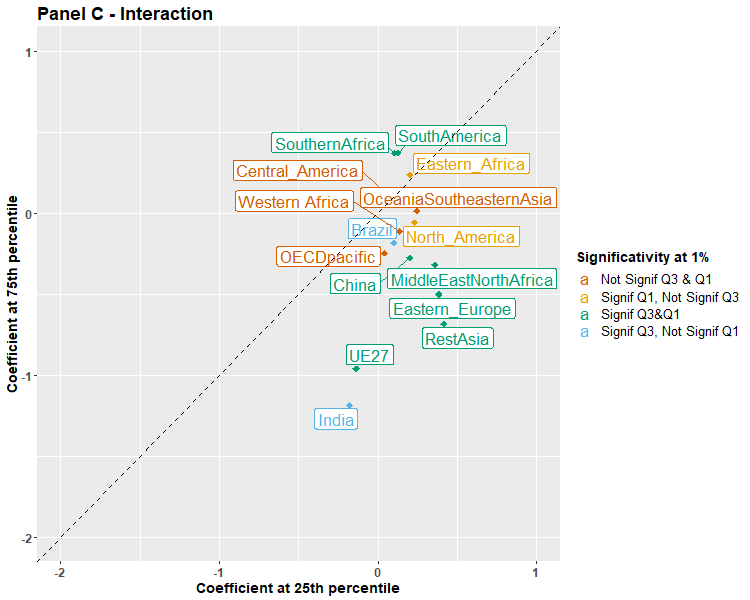


S14 Fig. R1 of quantile regression of two univariate models linking crop fraction to agricultural suitability (y-axis) and market influence (x-axis) using the Goldewijk et al. land use dataset (Klein Goldewijk et al., 2017). The highest R1 between the first and third quartiles of the distribution of crop fraction (Q1 and Q3) is reported. Values are computed at the third quartile for the regions labelled in black, at the first quartile for the regions labelled in blue, and at the third quartile regarding suitability (suit.) and the first quartile regarding accessibility (access.) for the region labelled in red


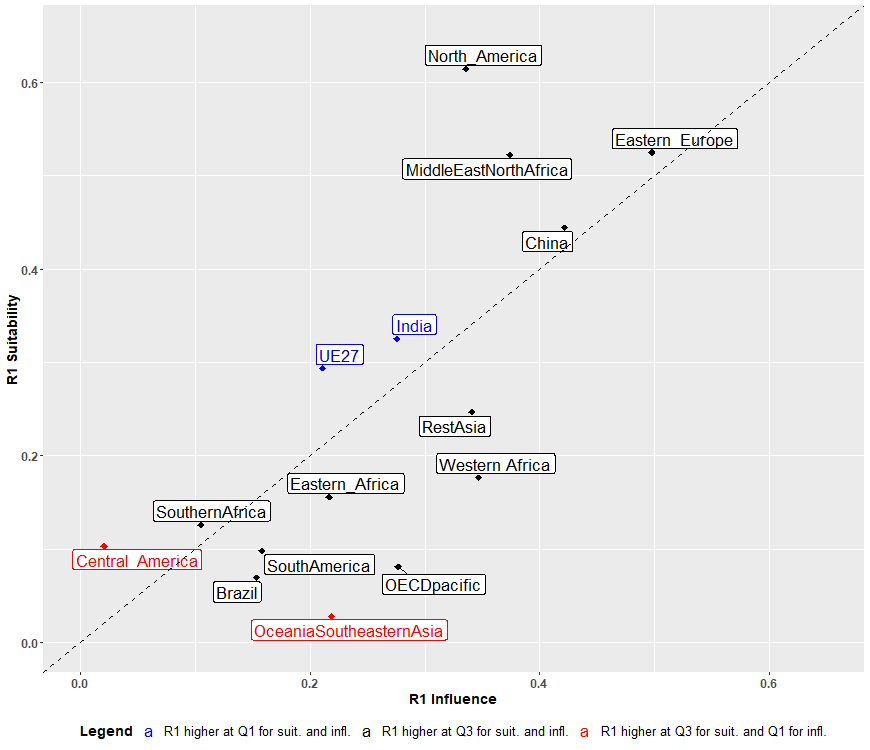


**S15 Fig. Zoomed-in maps of cropland fraction (upper), agricultural suitability (middle) and market accessibility (lower) in North America (left) and Brazil (right).**

| **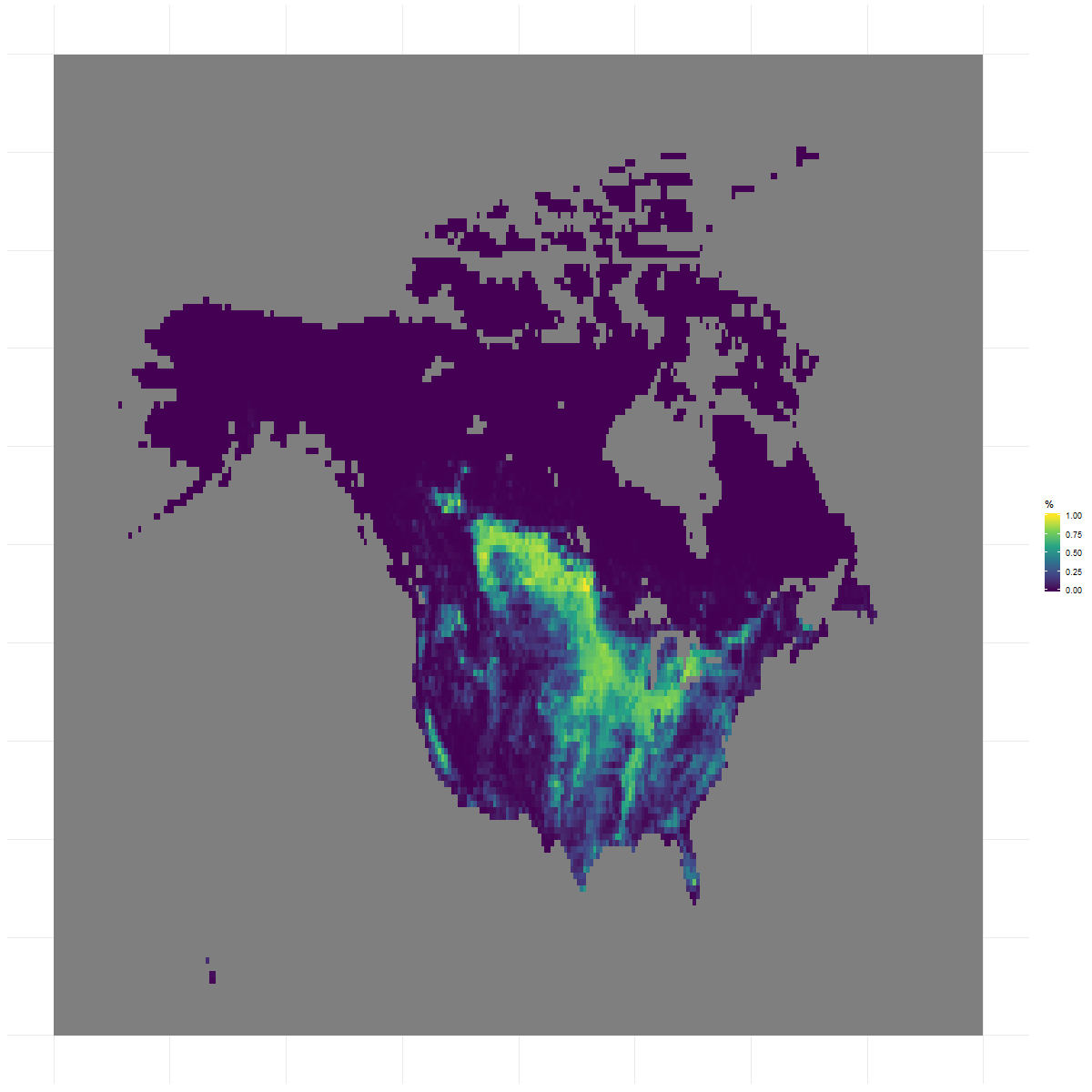** | **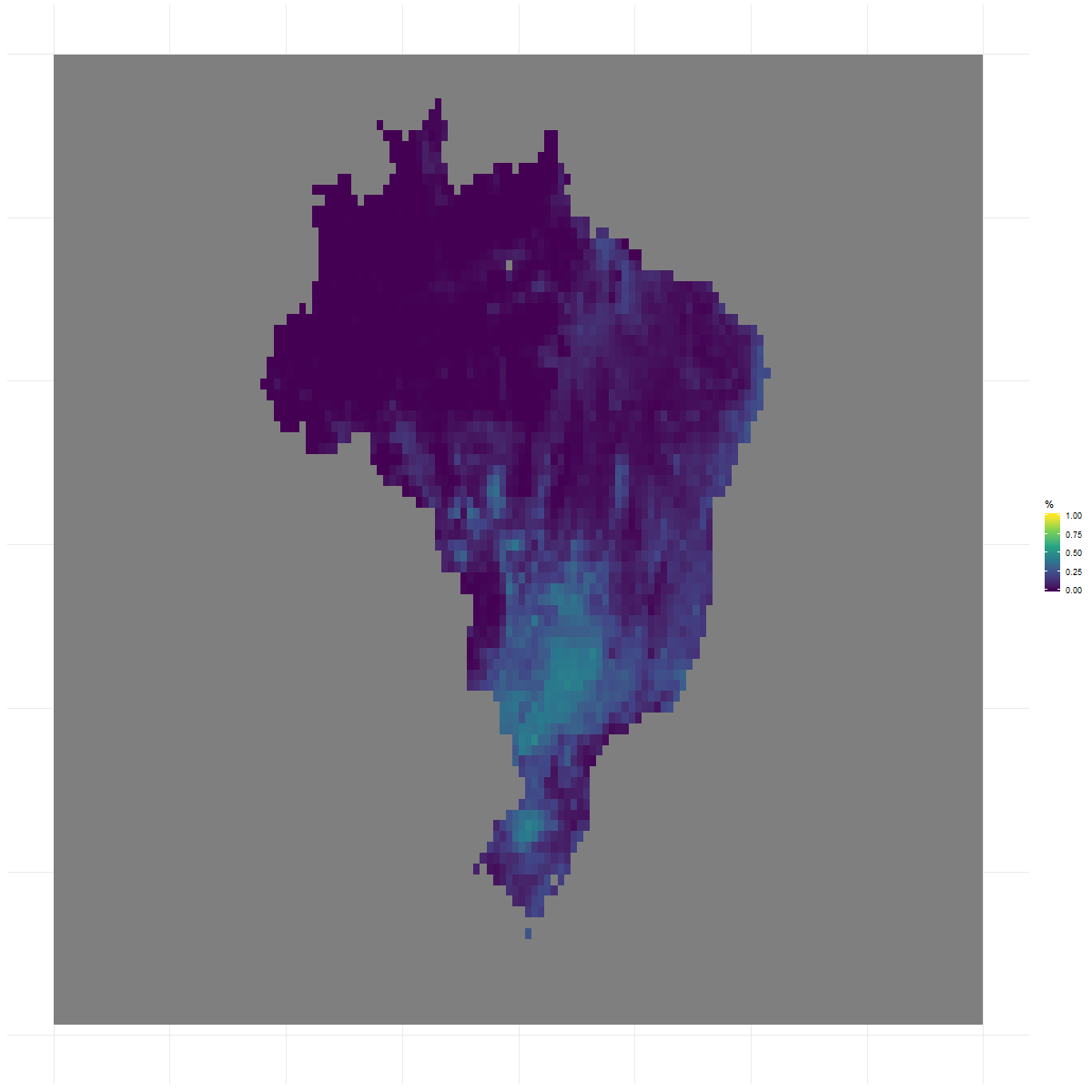** | **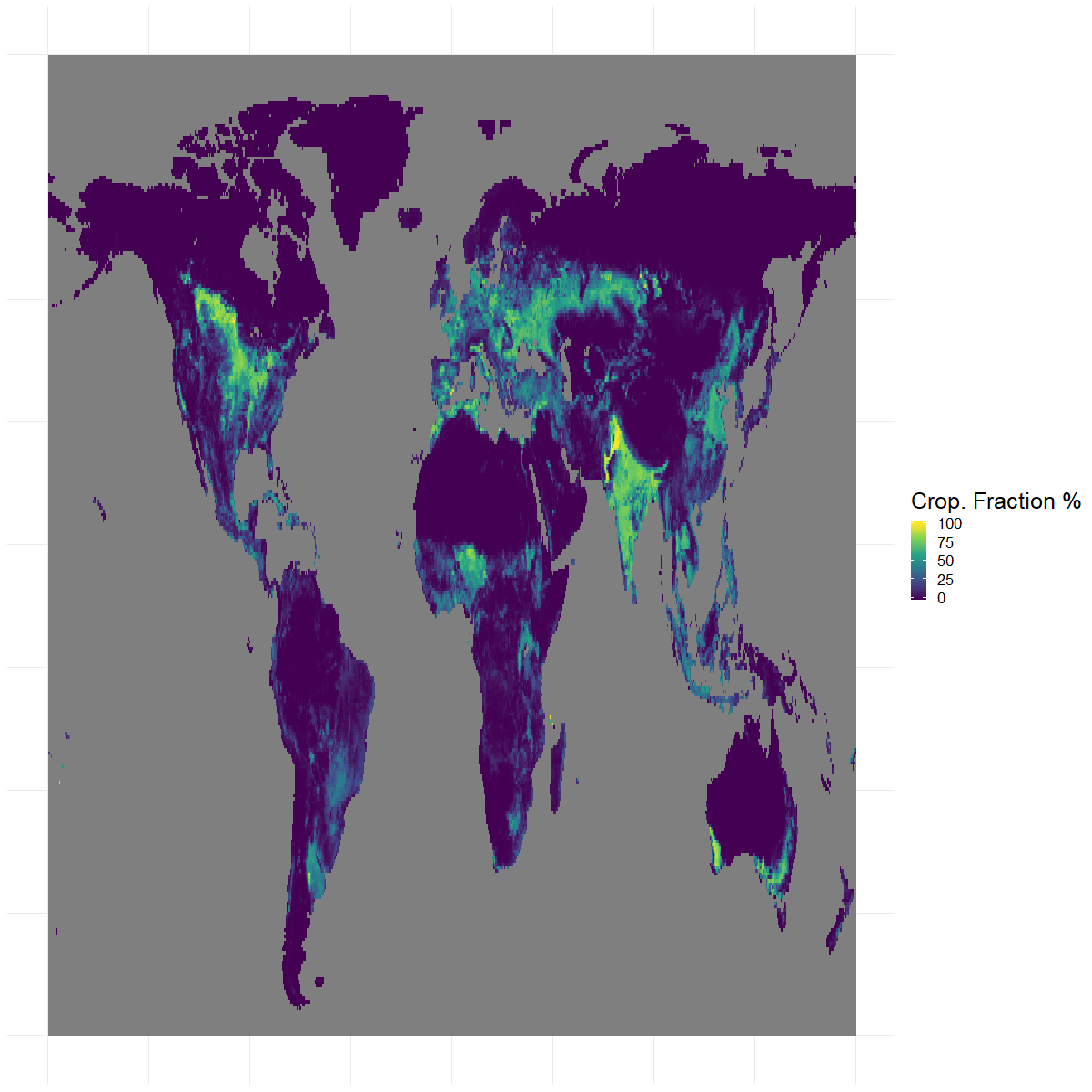** |
| --- | --- | --- |
| **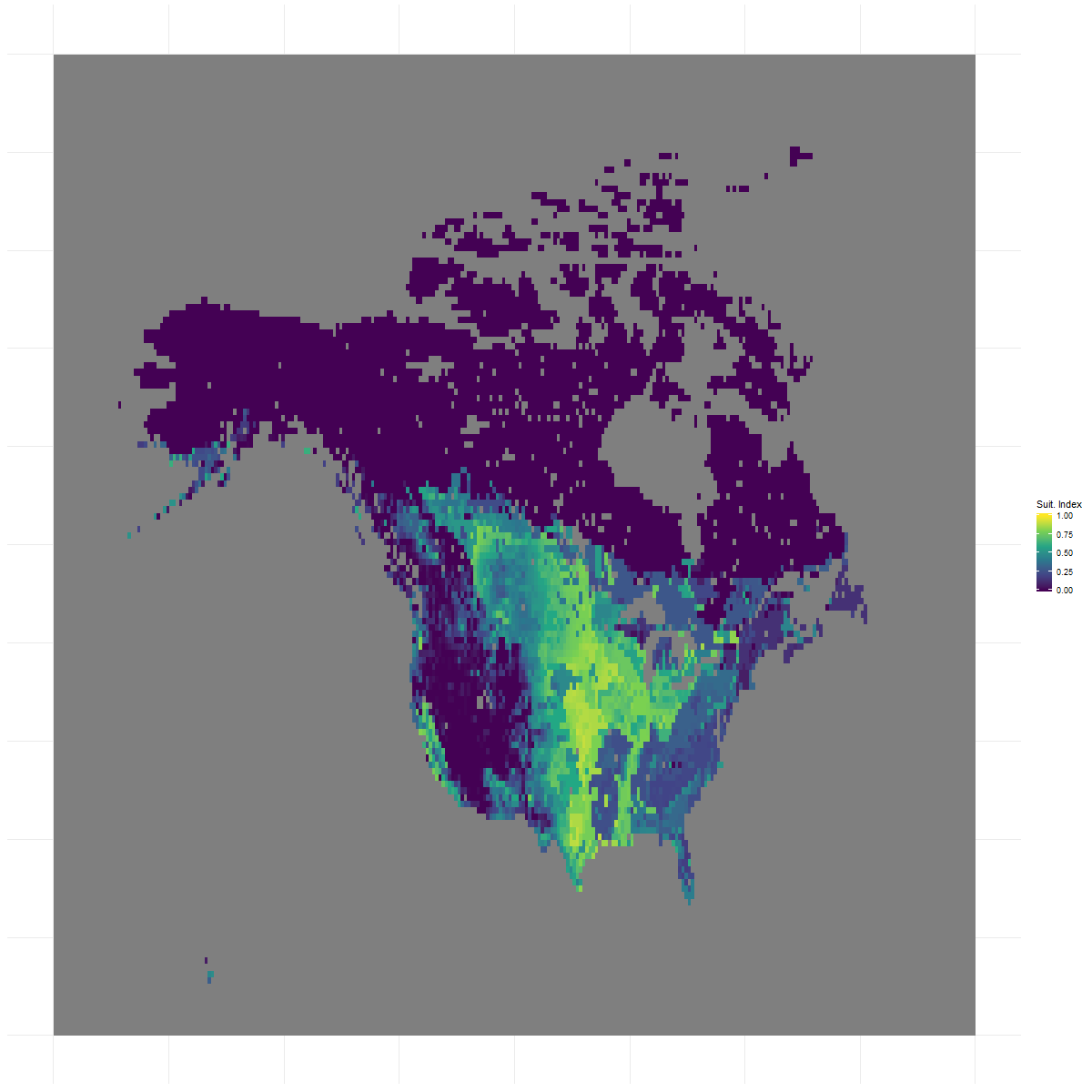** | **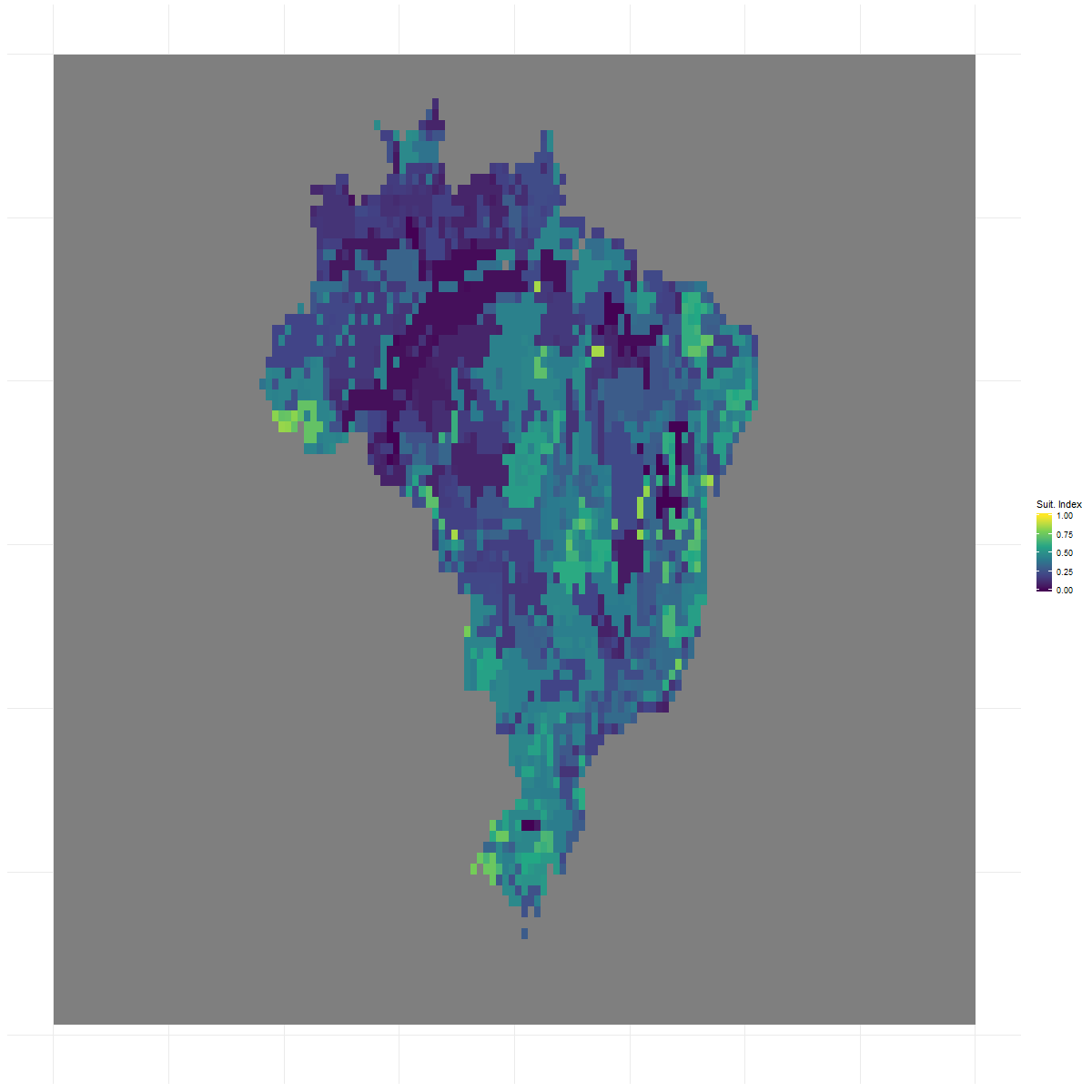** | **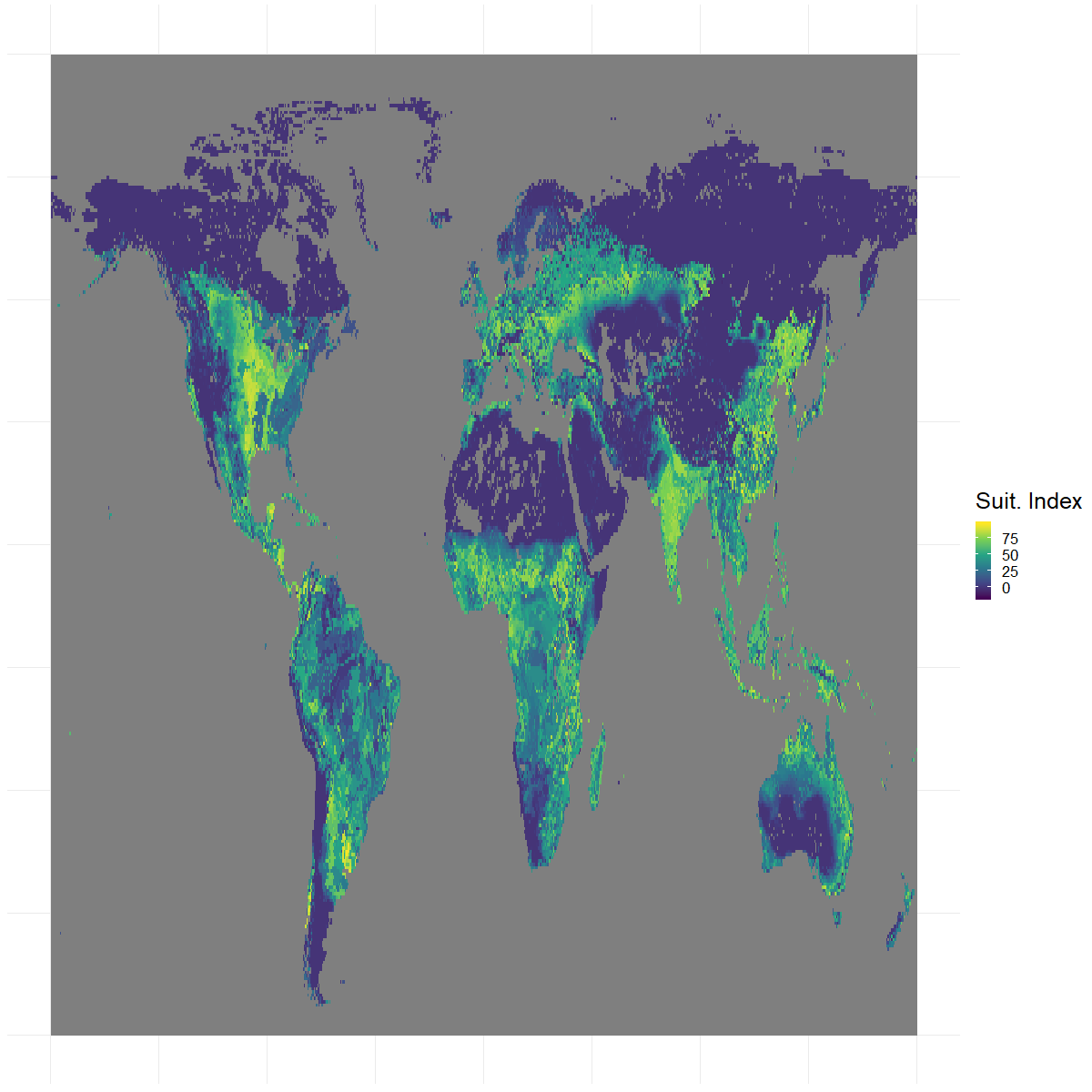** |
| **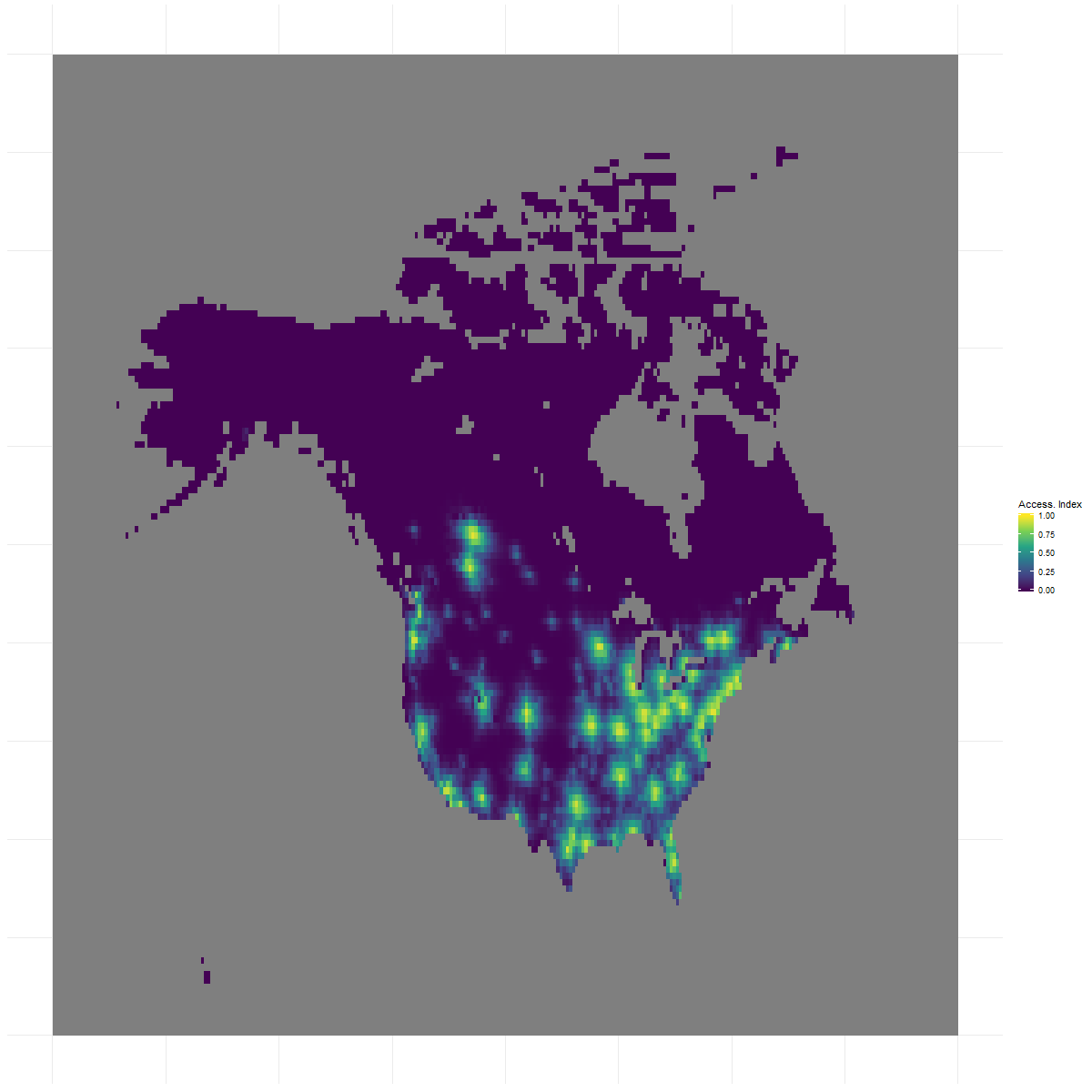** | **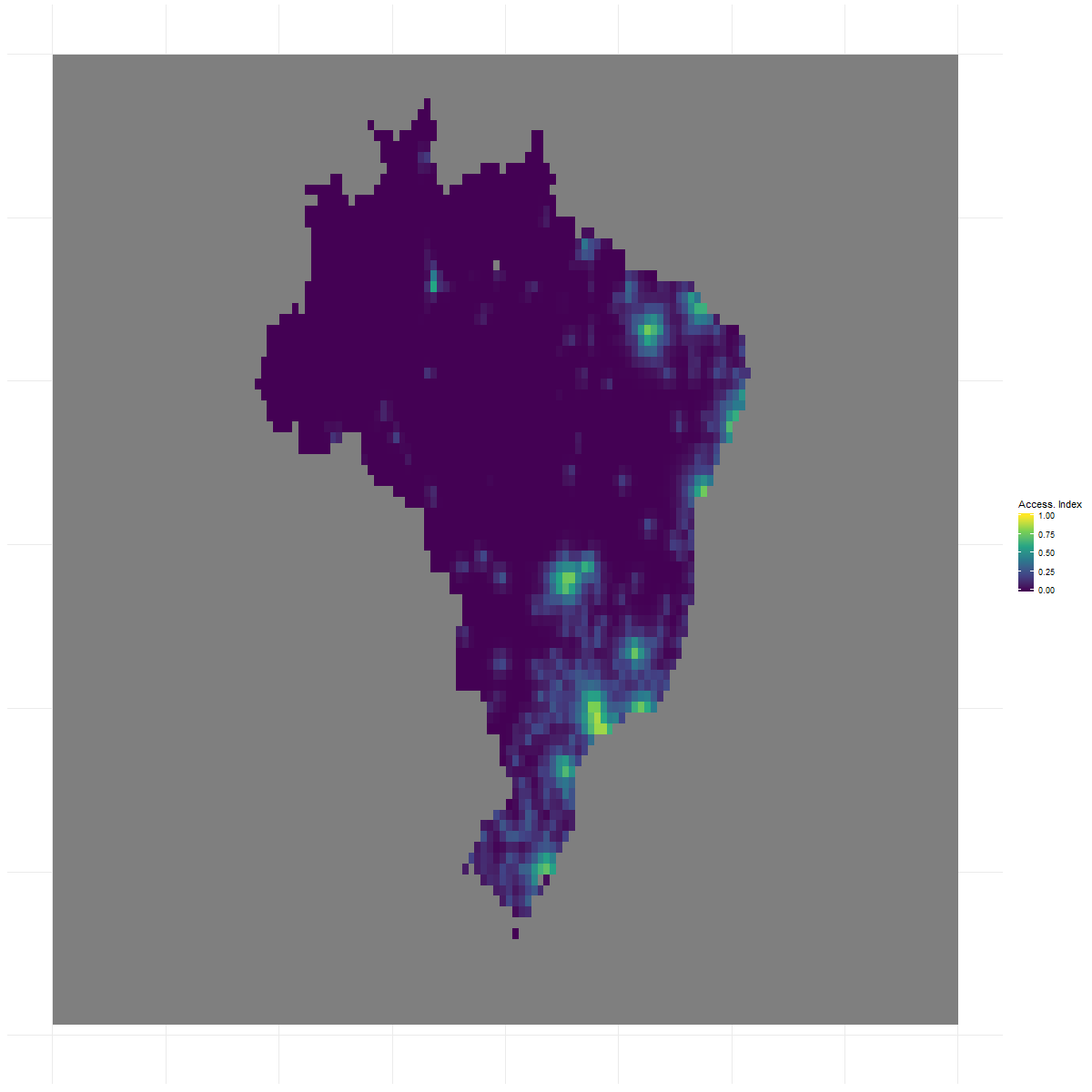** | 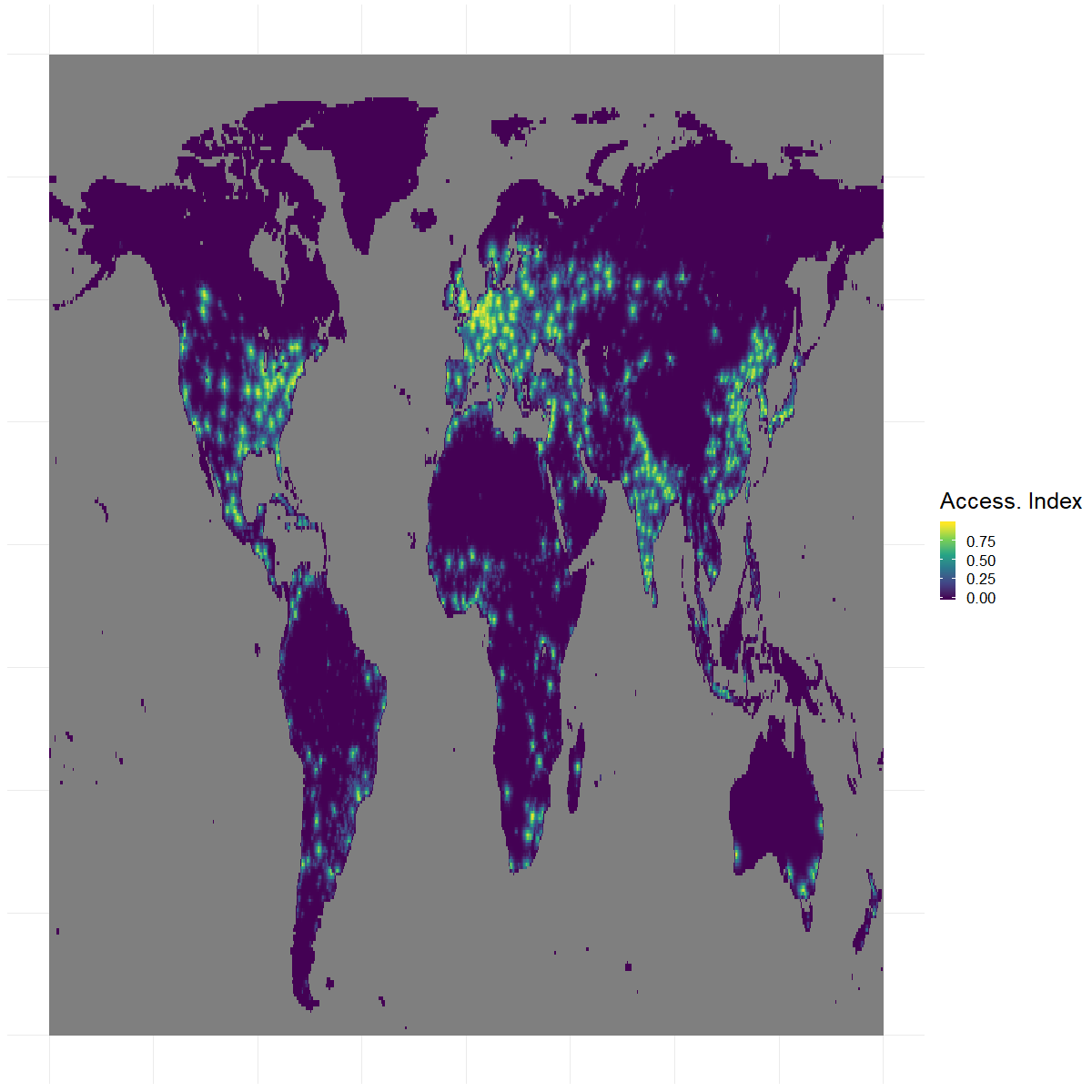 |
| The above maps show that crop fractions in North America can reach high values locally, especially in the Midwest and in the Mississipi Valley. On the contrary, crop fractions are overall lower in Brazil with more dispersed cultivated areas. The highest crop density is found in soybean producing areas in the State of Sao Paulo, Mato Grosso and Parana.  The distribution of agricultural suitability is also contrasted between the two regions. In North America, agricultural suitability is particularly high in the Middle-West and the Mississippi Valley while the distribution of agricultural suitability is more uniform in Brazil. We can see on the maps a good match between the cultivated areas and the most suitable land for agriculture in North America. This match is by contrast less evident in Brazil.  Market accessibility in Brazil is concentrated around the coastal cities and the capital Brazilia. In North America, accessibility is high in almost all coastal areas and also in some inland areas: near the cities bordering the Great Lakes (namely the triangle formed by Chicago, Detroit and Indianapolis) and in some regional capitals such as Denver. There is some match between cultivated areas and accessibility in North America, although the indicator is relatively imprecise regarding the corn and soybean exports that are shipped to Asian countries through inland barge transportation on the Mississippi rivers towards the Gulf of Mexico. In Brazil, there is a large accessibility area around Sao Paulo, close to the soybean production areas. However, many of the cultivated areas in Brazil are located in relatively inaccessible areas. | | |
|  | | |

**References**

Erb, K.-H., Gaube, V., Krausmann, F., Plutzar, C., Bondeau, A., Haberl, H., 2007. A comprehensive global 5 min resolution land-use data set for the year 2000 consistent with national census data. J. Land Use Sci. 2, 191–224. https://doi.org/10.1080/17474230701622981

Klein Goldewijk, K., Beusen, A., Doelman, J., Stehfest, E., 2017. Anthropogenic land use estimates for the Holocene – HYDE 3.2. Earth Syst. Sci. Data 9, 927–953. https://doi.org/10.5194/essd-9-927-2017

Verburg, P.H., Ellis, E.C., Letourneau, A., 2011. A global assessment of market accessibility and market influence for global environmental change studies. Environ. Res. Lett. 6, 034019.

Zabel, F., Putzenlechner, B., Mauser, W., 2014. Global Agricultural Land Resources – A High Resolution Suitability Evaluation and Its Perspectives until 2100 under Climate Change Conditions. PLOS ONE 9, e107522. https://doi.org/10.1371/journal.pone.0107522
