# Supplementary material for: Assessing whether the best land is cultivated first: A quantile analysis
Source: PLoS One. 2020 Dec 10;15(12):e0242222. doi: 10.1371/journal.pone.0242222 (PMC7728207; doi:10.1371/journal.pone.0242222)
Supplement: Attachment — Submitted filename: Response-Reviewers-R1-fin.docx [file pone.0242222.s002.docx]

Dear Editor,

We would like to thank the two reviewers for their careful reading of our paper. Their remarks were of great help to us to improve the paper. You will find in the table below our answers to the reviewers as well as the modifications made to the paper.

Best regards,

Thierry Brunelle, David Makowski

|  | Reviewer's comments | Authors'responses | Changes made to the paper |
| --- | --- | --- | --- |
| R1.1 | I am not entirely clear why the authors chose to use the HYDE database as a source for cropland areas, even though the fact that they come to similar results when using another dataset (Erb 2007) is reassuring. HYDE’s purpose being to reconstitute broad trends of land use over thousands of years, I am not sure its baseline map is as accurate as others that are available, such as for example the LUGE lab’s maps of cropland and pasture areas (available at http://www.earthstat.org/cropland-pasture-area-2000/), which is available at 5 minutes resolution. If the authors think that HYDE is the best choice of data in this case, perhaps a short justification would be in order. Also, the authors say the map is for 2017 but HYDE website says the current map is for 2000 and the HYDE 3.2.1. release note says that the maps go up to 2015. It would be good to clarify this (and confirm that it is indeed a map of cover and not a simulation that is being used in this analysis). | The HYDE database is an accurate dataset. It combines national statistics for different land use categories from FAO for the period 1960–2015, subnational levels statistics and spatially explicit depiction of land cover from the ESA Land Cover consortium maps for the year 2010. We choose the HYDE dataset over the Ramankutty et al. dataset because it is more recent and provides information on infrastructure areas which are needed in our study. The HYDE 3.2.1 database includes updated maps for the years 2016 and 2017. The data are available at : ftp://ftp.pbl.nl/hyde/hyde3.2/ | l.160-163: HYDE 3.2.1 combines national statistics for different land use categories from FAO for the period 1960–2015, subnational levels statistics and spatially explicit depiction of land cover from the ESA Land Cover consortium maps for the year 2010. Data can be found at ftp://ftp.pbl.nl/hyde/hyde3.2/ |
| R1.2 | Related to this, a more recent and possibly more accurate accessibility dataset might be worth considering: https://malariaatlas.org/research-project/accessibility_to_cities/ (published as doi:10.1038/nature25181). This also has a high resolution, of 1x1km2. | The dataset on market accessibility from Verburg et al. is based on an index computed from estimated travel times to cities. This index is calculated using a specific methodology distinguishing large and medium size cities, as well as large maritime ports. the Verburg et al. index could be updated by using the Weiss et al. data. However, carrying out such calculation is out of the scope of the paper. |  |
| R1.3 | Finally, the paper could explain better the selection and composition of the agricultural suitability index, considering the importance of that variable for the analysis. The authors say that “Global agricultural suitability is measured using an index reflecting the climatic, soil and topographical conditions necessary to grow the 16 most important food and energy crops” (l.153-155). The mention of 16 crops suggest that an index was built somehow from 16 suitability maps, begging the question of how. After looking up the source (https://zenodo.org/record/3748350) I realize that “The agricultural suitability represents for each pixel the maximum suitability value of the considered 16 plants”, but clarifying this in the paper would be better. As well, a few words about why this index is superior to others (e.g. the FAO GAEZ) would be welcome. | Thank you for this suggestion. We now mention in the revised paper that the agricultural suitability index indicates the maximum suitability value across the 16 crop species considered in each pixel. This index is strongly correlated with the GAEZ, as shown in Zabel et al. (2014). As the Zabel’s index is more recent, this index was chosen in our study. | l.165-168: This index represents for each pixel the maximum suitability value across the 16 crop species. This index is strongly correlated with the Global Agro-Ecological Zones index. As the Zabel’s index is more recent, this index was chosen in our study. Data can be found at https://zenodo.org/record/3748350#.XzpPjegzbIU |
| R1.4 | Spatial resolution: What is the rationale for the use of a 30 arc-min resolution? Unless I misunderstood, all datasets are available at a higher resolution (HYDE AND Verburg et al. at 5 arc-min, Zabel et al. at 30 arcsec), so I’m not sure why the analyses are run at 30 arc-min especially since it seems to cause some minor issues (l. 179 and following). Related to that, lines 181-2, it would be easier to understand if the granularity were expressed in terms of resolution (what do “two digits after the decimal point” represent?). | The 30 arc-min is the standard resolution for most spatially explicit global economic land-use assessments (see for instance Globiom or MAgPIE models). There is a misunderstanding concerning the interpretation of the term “granularity”. In our analysis, the granularity reflects the number of decimal place and not the spatial resolution. We make that clearer in the paper. Working at a 30 arc-min resolution offer important practical advantages. Several aspects of our analysis are computationally intensive (e.g., testing for spatial autocorrelation and calculating the theoretical cropland distribution). Conducting these analyses at a 5 arc-min resolution with 36 times bigger matrices would require very long computation time without clear advantages in terms of precision |  |
| R1.5 | When mapping the potential crop distribution, we don’t actually know the composition of crops that would be possible in that distribution: maybe it is theoretically possible to cultivate the same area of crops in that area; but is it possible to cultivate the same crop mix? The lumping of all crops together masks the fact that reaching the potential distribution would likely imply a significant restructuring of agricultural production. I don’t think that is a fatal flaw but I think it warrants some discussion in the paper. | That’s indeed an important point. We add a comment on this issue in the discussion. | l.510 to 518: In this study, we assume that the actual crop mix is close to the optimal one (i.e., the crop mix showing the highest suitability in a given grid cell). This seems a reasonable assumption because farmers generally tend to grow the species that are best adapted to their environment, i.e., to local climatic, soil and topographical conditions. In some cases, humans have nevertheless been able to shape their environment, through irrigation, drainage or terracing, to make it more suitable for agriculture. This may explain deviations from a perfectly efficient distribution (i.e., where all crops would be on the best land). Also, climate change may make the choice of crops more complex, leading possibly to larger discrepancies between the actual and optimal crop mix. |
| R1.6 | there may be political reasons that prevent the cultivation of some areas. Or, importantly, other uses that are not taken into account here (e.g., livestock herding, forestry) are competing with croplands. I think that the question of land competition is important to discuss here as cropland allocation doesn’t occur in a vacuum, so optimal cropland allocation may not mean optimal land allocation. Without such a contextualization, recommendations like those on lines 437-446 seem to come out of the blue | True. We already mention the possible political barriers to the efficient use of land l.464-469. We add more details on this issue in the revised manuscript. | l. 489-494: Making land allocation more efficient also implies that it is possible to substitute different types of land uses, such as croplands, pastures and forest areas. However, the level of substitutability between different types of land uses depends on the interplay of many parameters, including the cultural factors, the type of actors involved, and local agricultural and environmental regulations 40–42. For example, important complementarities between land use types (e.g., forest vs. cropland) in mosaic systems can impede the transition towards more efficient crop land allocation. |
| R1.7 | In the left-hand side of the diagram, it wasn’t immediately clear to me that the divisions within each quantile were different parcels. While this becomes clear by reading the text, I think that adding a visual legend showing quantile, parcel, and cultivated areas (with arrows and text) would help make it more intuitive. | done |  |
| R1.8 | I wonder if there would be a way to shade or hash that left part in such a way as to represent the theoretical distribution? That would provide another visual point of comparison. Just a suggestion, not a request. | We tried to hatch a theoretical distribution but this tends to overload the graph especially in case 4 with higher crop density. |  |
| R1.9 | I wonder if the regression curves are useful here. That plot is not used in the rest of the paper, so it feels a bit like we’re putting effort into understanding a plot for nothing | This plot is indeed not used in the rest of the paper. However, this graphic plays a pedagogical rule and is useful to help readers understanding the basics of quantile regression. |  |
| R1.10 | I wonder if you could comment a bit on the role of agglomeration economies | Agglomeration economies manifest themselves through spatial autocorrelation. This topic is covered in a paragraph starting at l. 240 in the Material and methods section |  |
| R1.11 | Figure 3: The shadings are a little confusing. I would suggest displaying both series of plots instead (accessibility and suitability). | Distributions over quartiles of land suitability and over quartiles of market accessibility are both available in SI (Fig. S1 and S2). For the figures to be relevant, both information need to be on the plot so we could have information of how much land is there that are both suitable and accessible. We did not find any other option than using shadings. |  |
| R1.12 | Figure 4: Do the colours have a meaning? If not it might be good to either include these same colours in the theoretical example in figure 1, or to revert to black and white. | The colors do not have any meaning. We removed it. |  |
| R1.13 | Figure 5: the labels are a bit cluttered, especially in panel A. Is there a way to avoid that the lines go over the labels? | Thanks for noting this. We use now short labels to avoid that the labels cover the dots. |  |
| R1.14 | Map: I think it would be good to have a zoomed-in map showing an example of how accessibility and suitability quantiles are distributed in a real-life territory. This would help grasp the concept better. | We add zoomed-in maps of cropland fractions, agricultural suitability and market accessibility in Brazil and North America in SI. | Comments below the zoomed-in maps :  The maps above show that crop fractions in North America can reach high values locally, especially in the Midwest and in the Mississipi Valley. On the contrary, crop fractions are overall lower in Brazil with more dispersed cultivated areas. The highest crop density is found in soybean producing areas in the State of Sao Paulo, Mato Grosso and Parana.  The distribution of agricultural suitability is also contrasted between the two regions. In North America, agricultural suitability is particularly high in the Middle-West and the Mississippi Valley while the distribution of agricultural suitability is more uniform in Brazil. We can see on the maps a good match between the cultivated areas and the most suitable land for agriculture in North America. This match is by contrast less evident in Brazil.  Market accessibility in Brazil is concentrated around the coastal cities and the capital Brazilia. In North America, accessibility is high in almost all coastal areas and also in some inland areas: near the cities bordering the Great Lakes (namely the triangle formed by Chicago, Detroit and Indianapolis) and in some regional capitals such as Denver. There is some match between cultivated areas and accessibility in North America, although the indicator is relatively imprecise regarding the corn and soybean exports that are shipped to Asian countries through inland barge transportation on the Mississippi rivers towards the Gulf of Mexico. In Brazil, there is a large accessibility area around Sao Paulo, close to the soybean production areas. However, many of the cultivated areas in Brazil are located in relatively inaccessible areas. |
| R1.15 | l.124 Typo: should be “a given parcel”. | done |  |
| R1.16 | l. 175 and throughout: I would suggest saying “Middle East and North Africa”, “Oceania and South-Eastern Asia” | done |  |
| R1.17 | l. 336-338: “However, in Eastern Europe, this positive effect can be at least partially offset by a strong negative and significant interaction between suitability and accessibility”: The plot indicates that India, Brazil, and RestAsia also have strong negative interactions. Why are they not included here? | True. We corrected that. | l. 359-361: However, in Eastern Europe, India, Rest of Asia and Brazil, this positive effect can be partially offset by a strong negative and significant interaction between suitability and accessibility |
| R1.18 | l. 453: “market accessibility, which is sometimes overlooked in land-use studies”: that is a surprising statement to me, as it seems that accessibility is the one thing that most land use scholars all agree is important. There may be some studies that don’t take it into account but this suggests that it’s being ignored more systematically which I don’t think is accurate. | Global economic land-use models are frequently coupled to vegetation model informing on agricultural suitability (usually through climatic potential yields), but the incorporation of market accessibility data is much less common. To our knowledge, out of the ten models or so involved in the integrated assessment communities, only two incorporate data on market accessibility (IMAGE and GLOBIOM). |  |
|  |  |  |  |
| R2.1 | I encourage the authors to dedicate some discussion to the use and application of von Thunen and ricardo in the recent literature. | We added some precisions on the use and application of classical rent theories l.58-62 (see response to comment R2.4). We also bring additional information in the discussion regarding the implications and limitations of our results regarding the theories currently used in the field (forest transition, abnormal rent, Boserup's induced intensification) | l. 455-469: It is noteworthy that the best fits are found in regions with a long history of intensive agricultural settlement - North America, Middle East, Eastern Europe, China, India and European Union - while the poorest fits are found in regions where the agricultural frontier remains active - especially Brazil and Oceania South Eastern Asia. This finding is consistent with the hypothesis of a process of gradual optimization of land allocation through learning described by Mather and Needle in the forest transition theory. […] Our results are also consistent with the findings of several authors regarding the presence of increasing marginal returns in areas of agricultural expansion (Cipolla, 1994; Richards and Arima, 2018) and support Di Tella's abnormal rent theory distinguishing between frontiers in equilibrium, where the price equals the average cost (zero profit) with a possibility of differential rent formation as one moves away from the frontier, and frontiers in disequilibrium with a possibility of positive profit and increasing returns.   l. 518 – 527: Finally, this analysis does not account for cropping intensity (i.e., the fraction of the cultivated area that is harvested). In doing so, we cannot conclude about Ester Boserup's critique of rent theories. This critique states that rent theories are based on an “oversimplified conception” of the agricultural system distinguishing between cultivated and uncultivated land, while landscapes are actually shaped by a continuum of land types that differ in their frequency of cropping^44^. The two views may be however not entirely divergent from a land use perspective. One can certainly think that there is a link between density of cultivated areas and cropping intensity. Low densities may be a signal of long fallows, while high densities are usually associated with annual or multiple cropping. This hypothesis, which remains to be confirmed, would be a way of reconciling the two sides. |
| R2.2 | I encourage the authors to streamline their reported statistics, and revise their graphics for readability and interpretation. | Graphics have been improved for readability and interpretation (see response to reviewers R1.7 and R1.12). |  |
| R2.3 | I encourage you to offer more information on the data inputs. This could be addressed with limited text in the manuscript and more detailed information in the supplementary materials. Ideally, you might also provide your code and datasets. Maps of the spatial data would also be useful. | We add further information on the data inputs (see response to reviewers R1.1, R1.3 and see opposite), with links to data repositories. Maps of crop fractions, agricultural suitability and market accessibility have been added in SI (see Fig. S15, 16, 17 and 18). | l.171 to 175: Data can be found at http://www.ivm.vu.nl/en/Organisation/departments/spatial-analysis-decision-support/Market_Influence_Data/index.aspx. Maps of cropland fraction, agricultural suitability and market accessibility are shown on Fig. S15, S16 and S17 in SI as well as zoomed-in maps for North America and Brazil on Fig. S18. |
| R2.4 | ‘Classical land rent theories imply that the best land is cultivated first.‘  This sentence could be re-written or dropped. Rent theory states that a location will be used for its most economically advantageous use. Generally speaking, this relates to the marginal returns to the use of labor and capital; while this usually translates to ‘the best land is used first’ this is not always the case. There are also tremendous questions here about what is meant by ‘best’. This article explicitly engages with this issue, but, if anything, it seems to highlight that the definition of best is hardly as clear as it is presented in this initial sentence. | The term "best" refers to the qualities of the land in terms of agricultural suitability and accessibility to markets. We make that clearer in the paper l. 72-73. Rent theories as originally formulated are based on the "intrinsic" qualities of the land. As explicitly mentioned by Ricardo: "The most fertile, and most favorably situated, land will be first cultivated". This principle is still in use in modern land use assessment through, e.g., the coupling with vegetation model informing on the agronomic qualities of land. We added some details on this l.58-62. Several new references have also been added (You et al., Havlik et al.). | l.58-63: According to these theories, land is assumed to be cultivated gradually in descending order according to its quality and its distance from the market, the best land being cultivated first, to quote Ricardo: "The most fertile, and most favorably situated, land will be first cultivated". Today, these theories are still directly applied in some land use assessments^18,19^. Many global land use models are rooted in classical rent theories by allocating land according to a profit function that depends on the intrinsic qualities of land provided by vegetation models (usually in terms of climatic potential yields)^20–22^ or based on index of agricultural suitability^23^ .  l.73: we provide a comprehensive empirical analysis of the effects of agricultural suitability and market accessibility, two major features characterizing the “best land” […] |
| R2.5 | L52-53. Add citations for this sentence. | done |  |
| R2.6 | L79. I’m wondering how you change the suitability of land. How often does that happen? (more info is needed to understand how you classified suitability, etc.). A map of agricultural suitability here would be very helpful. | We do not change the values of agricultural suitability. A map of agricultural suitability is added in SI. |  |
| R2.7 | L88-89 There is a decent case to make that regulations and ‘types of actors’ are elements of suitability; the former, especially, for land suitability. | This is not in line with the definition of suitability considered in this paper. Here, land suitability is defined based on environmental characteristics (soil and climate), not based on sociological characteristics. |  |
| R2.8 | Figure 1: the use of red as null squared at left, then as the histogram fill in the middle is confusing. In general, I find this graphic to be quite confusing. | The red color refers to the cultivated fraction within a given plot, not to uncultivated areas. This is directly related to the histogram filled in the same color. We made this point clearer in the revised version of Figure 1 where new explanatory texts were added. | See new figure 1. |
| R2.9 | Pages-6-7. I’m not sure I fully understand what the authors are trying to say here (as well as in figure 1). Perhaps the main point of these spatial schematics is this: ‘. Cropland can be preferentially distributed on the best land either by allocating crops to the least densely cultivated areas (better allocation) or by concentrating more crops on already densely cultivated areas (higher intensity) or both.’ But if so, I’m not following as to how density (given the article so far) should factor into the preferential distribution of agricultural land? Density is hardly a factor in classic rent theory (although some might say that this is a weakness…). | It is true that density is not a factor of the classic rent theory. However, these theories have geographical implications when considering cropland allocation on grid cells. The purpose of this section is to clarify these implications. Since the work is subsequently done with spatially explicit gridded data, this is a necessary pre-requisite. We clarify that in the paper. | l.91-94: The spatially explicit data sets that are currently available provide information on the cultivated fraction at the grid cell scale. However, we cannot directly infer from the formulation of rent theories their implications on this variable. To clarify this point, we show four contrasted patterns of cropland allocation on Fig. 1.  l.147-150: Fig. 1 summarizes the general approach adopted in this paper: we start from gridded data from which we derive crop distributions over land qualities (Q1-Q4). We then express these distributions as boxplots of cultivated fractions, and estimate the effect of land qualities for different quantiles of fractional crop coverage. |
| R2.10 | L150. ‘beginning of the 20th century…’ beginning? Or end? | Thanks for noting this. That's in fact in the beginning of the 21th century. This was corrected. |  |
| R2.11 | L175. It would be useful here to see a map of suitability and access. It is surprising to see that 9% of EE is classified as unsuitable (where?). At the same time, one might assume that much of South America might be unsuitable, although in percent terms the relative quantity might be small. | We added a map of cropland fractions, agricultural suitability and market accessibility in SI (see Fig. 15, 16, 17, 18) |  |
| R2.12 | L240. I’m not understanding why the authors are reporting that market access and suitability are spatially correlated. How could they be anything but? | We agree that the Moran test is not a key information. This is why we put it in SI. The spatial autocorrelation of market access and agricultural suitability was indeed expected, due to the similarities of several grid cells located in the same areas. The Moran test simply confirms that we do have significant spatial autocorrelation in all regions. |  |
| R2.13 | L275. Why do the authors think that NA favors suitable land? Is it luck? Is it better data (on suitability)? Is it better transportation systems? | The United States is a “textbook” case of gradual optimization of land allocation. In the US, crops were originally cultivated in relatively unsuitable land on the East Coast and in the Appalachian foothills (Boussard, 1987 ; Peet, 1969, Mazoyer and Roudart; 2002). The development of rail transport made it possible to exploit the fertile lands located in the Midwest and to export agricultural products to Western Europe.  Added references: Mazoyer, M., Roudart, L., 2002. Histoire des agricultures du monde: du néolithique à la crise contemporaine. Éditions du Seuil. Peet, J.R., 1969. The Spatial Expansion of Commercial Agriculture in the Nineteenth Century: A Von Thunen Interpretation. Econ. Geogr. 45, 283–301. https://doi.org/10.2307/142666 | l.460 to 464: The United States is a “textbook” case of gradual optimization of land allocation. In the US, crop allocation were originally cultivated based in relatively unsuitable land on the East Coast and in the Appalachian foothills 15,38,39. The development of rail transport made it possible to exploit the fertile lands located in the Midwest and to export agricultural products to Western Europe. |
