# Supplementary material for: Assessing whether the best land is cultivated first: A quantile analysis
Source: PLoS One. 2020 Dec 10;15(12):e0242222. doi: 10.1371/journal.pone.0242222 (PMC7728207; doi:10.1371/journal.pone.0242222)
Supplement: Attachment — Submitted filename: Response-Reviewers-R2-fin.docx [file pone.0242222.s003.docx]

Dear Editor,

We would like to thank the two reviewers for their feedback on our paper. You will find in the table below our answers to the reviewer 2 as well as the modifications made to the paper.

Best regards,

Thierry Brunelle, David Makowski

| L39. I would hesitate to refer to sources 8-9 as foresight studies. I think that ‘projections’ or something similar is much better word choice. | done |
| --- | --- |
| L53. The wording ‘is allocated’ should be changed. This suggests that someone is doing the allocation, so to speak. This is presumably true for individual properties or land portfolios. But I don’t think that there are many scenarios where, outside of basic zoning laws, farmland use is allocated by land use planning commissions. Change the wording here to refer to influence rather than allocations. The former implies local decisions, whereas the latter implies some sort of authoritarian decision process. This use of allocated recurs throughout. Please adjust. | We agree that the word "allocated" implies that someone makes the allocation, but this does not imply anything about the type of decision making. An individual can allocate resources without any form of authoritian process. Nevertheless, in order to avoid any ambiguity, we have replaced the term "is allocated" by "is used". |
| L60 ‘Many global land use models are rooted in classical rent theories by allocating land according to a profit function that depends on the intrinsic qualities of land provided by vegetation models (usually in terms of climatic potential yields) or based on index of agricultural suitability.’ Global land use models are generally rooted in elasticities and responses to market possibilities or climate trends. Classical rent theory, of course, is implicit in these relationships. | True. We add in the paper the following comment (l.64): "Land supply elasticities are also generally used to determine land conversion rates in a given location. In this case, the elasticity is estimated based on assumptions derived from rent theories (Villoria et al., 2018)". |
| L.67 ‘However, at the local scale, several empirical studies show inconsistent responses to agricultural suitability and market accessibility across regions24. Although strong relationships have been observed between cultivated area and agricultural suitability and market accessibility in several European regions, some croplands in China were recently moved to less fertile areas in response to urbanization dynamics. This is very much aligned with classical rent theory, not inconsistent with it. | True. We removed the word "inconsistent" and we have modified the sentence l.68 as follows: "Several empirical studies at the local scale have investigated responses to agricultural suitability and market accessibility". |
| L69. Moreover, assessments carried out at the land system scale show contradictory effects of spatial determinants on land allocation24. I’m not sure what this sentence is trying to say, why it is included, or what effects that are referring to. | For more clarity, we have modified the sentence as follows (l.72-74): "At the land system level, several assessments reported ambiguous effects of some spatial determinants on land use, like for example a negative effect of market accessibility on agricultural land use. 25". |
| L101. The use of the term ‘best land’ continues to be problematic. Do they mean ‘optimal’ in terms of suitability and access? | We have clarified this point l. 59-60 that: "In this paper, we refer to the highest grades of land in terms of potential productivity, location suitability or both as “best land". And in lines 80-81: "In this paper, we refer to the areas with the highest suitability and/or accessibility indices as "best land". |
| L258. This paragraph can be deleted or moved to the SI. I’m not sure what it adds. (somewhat annoyingly, I raised this clearly in the initial review) | We agree that the Moran test does not bring much information. It is only a confirmation. That is why we have replaced the term "reveals" by "confirms". However, we do not wish to remove this paragraph as we think it is important to include an explanation on how spatial auto-correlation has been treated in the paper. |
| L455. The note on fits by region is quite interesting and begs more discussion. Presumably, it’s not only that land optimization has increased over time, but that transportation costs have also shifted with time. | Reduction in transportation cost is indeed a key driver of the optimization process. We clarify this point in the discussion l.475-478: "The reduction in transport costs resulting from the development of the railways, combined with the reduction in transatlantic freight rates, made it possible to cultivate the fertile lands of the Midwest and export part of the agricultural production to Western Europe." |
| L496. This discussion of ‘grand theories’ and the warning against oversimplification in their application is in many respects and oversimplification in the characterization of these theories. In effect, there is tremendous nuance in how the theory is applied and manifested in land economics. While I find the simplicity of this analysis to be useful for clarifying a widely used model, the authors at times show a simplicity in their knowledge of the field and how these theories are used. | We agree that the theory can be applied in different ways. Here the term "grand theory" is not pejorative. It refers to the distinction made by Meyfroidt et al. between middle-range theory and general theoretical frameworks. This distinction is very nuanced as it accepts the need for generalization. The expression "oversimplified conception" is not ours and refers to the Boserupian conception of the use of cultivated land on which we are unable to conclude. |
| Map figure. Please include a map of the suitability and access regions in the main text. | Done |
